# Comparison of oral Nano-Curcumin with oral prednisolone on oral lichen planus: a randomized double-blinded clinical trial

**DOI:** 10.1186/s12906-020-03128-7

**Published:** 2020-10-31

**Authors:** Seyed Javad Kia, Maryam Basirat, Tahereh Mortezaie, Mahdieh-Sadat Moosavi

**Affiliations:** 1 Dental sciences research center, Department of Oral and Maxillofacial Medicine, School of Dentistry, Guilan University of Medical Sciences, Rasht, Iran; 2 Dental Research Center, Dentistry Research Institute, Department of Oral and Maxillofacial Medicine, Faculty of Dentistry, Tehran University of Medical Sciences, Tehran, Iran

**Keywords:** Curcumin, Nanoparticles, Oral lichen Planus

## Abstract

**Background:**

Oral lichen planus (OLP) is a mucocutaneous autoimmune disease with T-cell mediation. Corticosteroids are considered as a first choice in OLP and should be used for a long period with a subsequent increase in dose since the disease has a chronic and recalcitrant nature. There have been efforts to use alternative therapies due to the Corticosteroid’s side effects. Curcumin is a non-toxic natural product with different effects on various oral diseases. It demonstrates antioxidant, anti-inflammatory, antimicrobial, and anticarcinogenic activities. It seems that Curcumin can be used as a proper alternative for Corticosteroid treatments. To overcome limitations in the bioavailability of Curcumin, the therapeutic effect of oral Nano-Curcumin was evaluated for the first time.

**Methods:**

Sixty OLP patients were included in this double-blinded randomized clinical trial. The patients were randomly divided into two groups and received either ‘Nano-Curcumin 80 mg’ or ‘Prednisolone 10 mg’ treatments for 1 month. The patients should take one capsule after having their breakfast. The VAS scale was used to analyze pain severity and burning sensation. To assess lesion size the Thongprasom scale was employed. Repeated measures and independent t-tests, as well as LSD paired-test, were used to analyze the data.

**Results:**

Data from 57 patients were analyzed. The level of pain, burning sensation, and OLP lesions decreased in both groups of Curcumin and Prednisolone and no statistically significant difference was observed between the two groups.

**Conclusion:**

Despite many studies conducted to find an effective approach for managing OLP, the results have often been unsatisfactory. In comparison with previous studies, current results clarify the importance of Nano-Curcumin bioavailability in therapeutic effects. Pain VAS and lesion size were decreased with oral Curcumin. The results have shown that oral Curcumin can be used as an alternative therapy for OLP in patients with the contraindicated Corticosteroids or should be used with caution. Oral Curcumin can be used in preventing the recurrence of OLP lesions after the treatment and initial control. Moreover, the amount of Curcumin dose is more important than its use duration in improving OLP.

**Trial registration:**

IRCT, IRCT20100101002950N5. Registered 9 February 2019, https://www.irct.ir/trial/36704.

## Background

Oral lichen planus (OLP) is a kind of chronic mucosal disease identified as an immune disorder [1]. There are several forms of OLP lesions, namely reticular, papular, plaque-like, bullous, erythematous (atrophic), and ulcerative [2]. There is a range of epidemiological researches. It has been estimated that the frequency of OLP would range from 0.55 to 2% [3, 4].

The commonest involvement site for OLP is the buccal mucosa. However, other oral cavity sites, such as labial mucosa, tongue, and gingiva, might be influenced as well [5]. OLP presentation is primarily connected with symptoms, ranging from a burning sensation to severe pain. This presentation barely remits extemporaneously [3, 5, 6]. Most patients suffering from OLP have periods of relapses and remissions. There is a growth in detectable clinical signs and symptoms within periods of exacerbation, which can be related to psychological disorders or stress [5, 7, 8].

The treatment options presently available focus on alleviating the symptoms and monitoring any possible dysplastic changes [9]. Given the lesion’s severity, different therapeutic modalities have been employed- either on their own or in combination- topically, intralesionally, or systemically. Using corticosteroids is the current acceptable mode of treatment [10]. These drugs should be used for a long period with a subsequent increase in dose since the disease has a chronic and recalcitrant nature. Nonetheless, the topical drugs bring about different side effects including thinning of the oral mucosa, secondary candidiasis, stomatopyrosis, and altered taste sensation. On the other hand, systemic steroids can lead to other side effects, such as suppression of the hypothalamic-pituitary axis, diarrhea, fluid retention, osteoporosis, hypertension, diabetes mellitus, and increased susceptibility to infection [11].

As a result, researchers have been continuously looking for a substitute natural or herbal drug to be taken as monotherapy or in combination with the first choice drugs [12]. These experts have been searching for drugs that could be taken in the treatment of lichen planus on a long-term basis with minimal side effects. This can help the specialists to control the disease and prevent the recurrence of the lesion. Curcumin is a non-toxic natural product with therapeutic effects on various oral diseases such as oral submucous fibrosis, leukoplakia, and Chemoradiotherapy-induced oral mucosal lesion [13, 14]. Scientists have categorized Curcumin as a natural phytochemical and active principle in turmeric, the ground powder of the rhizomes of *Curcuma longa*. Curcumin demonstrates antioxidant, anti-inflammatory, antimicrobial, and anticarcinogenic activities [15].

Furthermore, high doses of Curcumin would not be dangerous. The down-regulation of inflammatory transcription factors (e.g., nuclear factor-kappa B), enzymes (e.g., cyclooxygenase 2 and 5, lipoxygenase), and cytokines (e.g., TNF-α, IL-1, IL-6, IL-8) helps Curcumin to mediate its anti-inflammatory effects. Besides, the inhibition of free radicals and nitric oxide makes Curcumin produce its antioxidant effect [16]. Some previous studies have examined the effect of Curcumin on OLP. However, these studies have been accompanied by some limitations. In some studies, it has been used topically or systemically and with non-Nano-Curcumin form, which reduces its therapeutic effects due to Curcumin low bioavailability [2, 17, 18]. Another study has evaluated the effect of Curcumin in combination with Corticosteroids, which can continue to be limited in people with a contraindication on corticosteroid use [19].

Thus in the current study, the therapeutic effects of oral Nano-Curcumin in oral lichen planus are investigated for the first time. Curcumin nanoparticles have a greater absorption dose and bioavailability than Curcumin.

## Methods

*The current RCT report followed the standard checklist of CONSORT. This was a phase* 3 parallel clinical trial study. The patients with erosive and atrophic forms of OLP referred to the Oral Medicine Department *in 2018–2019 were considered as the study population.* The written informed consent was completed by the patients, and the study protocol was approved by the Ethics Committee of the University (IR.Gums.Rec.1397.295). The registration number of the clinical trial in a Primary Registry in the WHO Registry Network was IRCT20100101002950N5.

To determine the sample size, according to the repetition of sizes in 2 groups for each person, the effect of each person was considered. To do this, the formula was used to fit the repetitive sizes. Considering 4 repetitions, a correlation of 0.50, a statistical power of 95%, an error level of 0.05, and a standard deviation of 2.98 were all obtained from previous studies; the minimum sample size was 25 people for each group [20].

$$ n=\frac{2{\left({z}_{\frac{\alpha }{2}}+{z}_{\beta}\right)}^2{\sigma}^2\left(1+\left(m-1\right)\rho \right)}{md^2}=\frac{2{\left(1.96+1.64\right)}^2{2.98}^2\left(1+(3)0.5\right)}{4{(2.4)}^2}=24.98\cong 25 $$

The patient recruitment was shown in a CONSORT flow diagram. Sixty patients were examined, and patient characteristics containing personal and clinical data, such as age, sex, medical background, smoking habit, type and site of oral lesions, disease duration, type of treatment received earlier, as well as their pain or lesion severity, were recorded. The patients were then randomly (generated random numbers in Excel) divided into two groups.

OLP diagnosis was made by the modified WHO criteria (clinically the presence of bilateral lesions with white, reticular/popular components, and histologically the presence of liquefaction degeneration of the basal layer, and band-like infiltration of mononuclear inflammatory cells in the superficial connective tissue) [21]. Exclusion criteria were as follows: pregnancy; lactation; patients taking Corticosteroids; patients with elevated liver enzymes taking anticoagulants or anti-fungal drugs such as Warfarin (Curcumin has an inhibitory effect on platelet aggregation), orthodontic treatment, gastric ulcer, duodenal ulcer, and gallstone (Curcumin may cause an upset stomach and gallbladder contraction); the presence of any malignant or viral infection in the mouth; the presence of dysplasia in histopathology, receiving topical treatment for OLP within the last 2 weeks or systemic treatment for OLP within the last 4 weeks; taking azathioprine, cyclosporine, PUVA, UVA, or UVB within the last month; having allergies to Corticosteroids or herbal compounds, such as turmeric.

### Study groups

#### Group A

The 80 mg Curcumin capsule was prescribed. Nano-Curcumin capsules were provided by ExirNanoSina (a knowledge-based company). Its trade name is SinaCurcumin that contains 80 mg Curcumin in the Nano-Micellar Soft gel capsule. Oral nano-curcumin is available with two different dosages; 40 mg and 80 mg. According to the pilot study on OLP patients, 40 mg is not sufficient enough for symptoms relief, and based on the safety of 80 mg without any side effects, the 80 was chosen. The patients were told to take one capsule after having their breakfast.

#### Group B

The 10 mg Prednisolone was provided in capsules as group A. The patients should take one capsule after having their breakfast.

10–20 mg Prednisone and Prednisolone were taken daily for rather serious cases, and 35 mg Prednisone and Prednisolone were used daily for more serious cases within 2 weeks in order to treat OLP. Patients were treated with this standard protocol or/and topical steroids if they dropout.

In the present research, the 10 mg dose was consumed daily within 4 weeks. Since a dose of less than 7.5 mg Prednisone is physiologic (which is as strong as Prednisolone), tapering was done by taking a daily dose of 5 mg Prednisolone within 10 days.

### Data collection

This was a double-blind study. The random selection information was only available to a person who was not involved in the study and the required number of drugs for the two groups were counted and placed in an envelope for each patient; patients were unaware of the type of intervention. The necessary explanations for taking the medication for each patient were provided privately. The patient’s clinical examination, besides the measurement of their lesions and pain severity, was performed by an oral medicine specialist without knowing the grouping of patients. The examinations were done at the beginning of treatment and after its onset under the unit’s light within 1, 2, 4 weeks intervals and recorded in the patient’s datasheets. The patients’ pain severity was measured by VAS (Visual Analogue Scale). At each visit, the patient was asked for a degree of pain that ranged between 0 and 10. Grade 0 indicated painlessness and grade 10 represented the most severe kind of perceptible pain. Sterile calipers were employed to measure the lesions. Thongprasom scale was also employed. This scale was graded as follows:

- 0) There is no lesion and the person is not sick.
- 1) Mild white stretch marks, with no erythematous site.
- 2) White stretch marks with the atrophic site, with a size smaller than 1 cm^2^.
- 3) White stretch marks with the atrophic site, with a size bigger than 1 cm^2^.
- 4) White stretch marks with the erosive site, with a size smaller than 1 cm^2^.
- 5) White stretch marks with the erosive site, with a size bigger than 1 cm^2^.

### Statistical analysis

Due to the normal distribution of data, ANOVA with repeated measure test was used for statistical analysis. Friedman’s test was employed if the data was not normally distributed. All the tests were carried out at a 5% level using SPSS24.

## Results

Data from 57 patients (Group A, *n* = 29; Group B, *n* = 28) with oral lichen planus were analyzed in this study due to the lack of referral of 3 patients. There were no significant differences between the two groups for age and gender (*p*-value > 0.05). The basic characteristics of the studied groups were shown in Table 1.

**Table 1:** Basic characteristics in the studied groups

	Curcumin	Prednisolone	*P*-value
Female/Male ratio	25/4	23/5	0.274
Age (mean (SD))	51.86 (9.94)	53.67 (8.90)	0.533
Baseline VAS (mean (SD))	4.65 (3.39)	4.89 (3.34)	0.818
Baseline Lesion Size (mean (SD))	3.83 (1.17)	3.61 (0.98)	0.515

Table 2 presents a two-by-two inter-time comparison between the two groups. Based on the results, the mean VAS did not differ significantly only before and 1 week after the study (*p* = 0.348). At other times, the difference was significant, and the average VAS score decreased with increasing time. Table 3 provides the inter-time comparison in the two examined groups. According to the results, the VAS mean had a significant difference at all the examined times, and the VAS mean dropped with an increase in time. Table 4 shows a comparison of VAS means in the two groups at each of the examined times. According to the results, there was no significant difference at any of the examined times. The results of the test demonstrated that the VAS score followed a falling and meaningful change trend during the examined time (*p* < 0.001). However, this changing trend did not have a significant difference in the two groups (*p* = 0.428).

**Table 2:** A two-by-two inter-time VAS comparisons in Curcumin group

	Before the study	Week 1	Week 2	Week 4
Before the study	–	–	–	–
Week 1	0.348	–	–	–
Week 2	0.001	0.002	–	–
Week 4	< 0.001	< 0.001	< 0.001	–

**Table 3:** A two-by-two inter-time VAS comparisons in Prednisolone group

	Before the study	Week 1	Week 2	Week 4
Before the study	–	–	–	–
Week 1	0.042	–	–	–
Week 2	< 0.001	0.002	–	–
Week 4	< 0.001	< 0.001	0.001	–

**Table 4:** A comparison of VAS mean in the two groups at each of the examined times

Time	Before the study	Week 1	Week 2	Week 4
Test statistics	0.23	0.30	0.16	0.46
Significance	0.818	0.766	0.869	0.65

Table 5 shows inter-time comparisons of lesion size in the Curcumin group. According to the results, there were significant differences between all the examined times. Lesion size changes followed a falling trend. Table 6 presents inter-time comparisons of lesion size in the Prednisolone group. According to the results, there were significant differences between all the examined times. Lesion size changes followed a falling trend. Table 7 demonstrates a comparison summary of the lesion size means in the two groups at each of the examined times. Base on the results, there was no significant difference at any examined time.

**Table 5:** Inter-time Lesion Size comparisons in Curcumin group

	Before the study	Week 1	Week 2	Week 4
Before the study	–	–	–	–
Week 1	0.005	–	–	–
Week 2	< 0.001	< 0.001	–	–
Week 4	< 0.001	< 0.001	0.003	–

**Table 6:** Inter-time Lesion Size comparisons in Prednisolone group

	Before the study	Week 1	Week 2	Week 4
Before the study	–	–	–	–
Week 1	0.015	–	–	–
Week 2	< 0.001	< 0.001	–	–
Week 4	< 0.001	< 0.001	0.003	–

**Table 7:** A summary of lesion size mean comparison in the two groups at each of the examined times

Time	Before the study	Week 1	Week 2	Week 4
Test statistics	0.66	0.74	0.74	1.60
Significance	0.515	0.465	0.462	0.117

According to the results, lesion size changes followed a falling trend and were significant (*p* < 0.001). However, this changing trend did not have a significant difference in the two groups (*p* = 0.568).

Tables 8 and 9, showed inter-group comparisons between two groups. Based on the results, the level of pain, burning sensation, and OLP lesion decreased in both Curcumin and Prednisolone groups and there was no significant difference between them.

**Table 8:** Pain VAS means in the two groups at the examined times

Time	Group	Mean (Standard deviation)	Significance group’s time test statistics
Before the study	Curcumin	4.65 (3.39)	0.85
	Prednisolone	4.89 (3.34)	0.428
Week 1	Curcumin	4.38 (3.03)	
	Prednisolone	4.67 (3.45)	
Week 2	Curcumin	3.41 (2.74)	
	Prednisolone	3.28 (2.74)	
Week 4	Curcumin	2.69 (2.89)	
	Prednisolone	2.33 (2.03)	
Time test statistics		40.02	
Significance		< 0.001

**Table 9:** Lesion size means comparison in the two groups

Time	Group	Mean (Standard deviation)	Significance group’s time test statistics
Before the study	Curcumin	3.83 (1.17)	0.61
	Prednisolone	3.61 (0.98)	0.568
Week 1	Curcumin	3.48 (1.27)	
	Prednisolone	3.22 (1)	
Week 2	Curcumin	2.79 (1.15)	
	Prednisolone	2.56 (0.92)	
Week 4	Curcumin	2.34 (1.14)	
	Prednisolone	1.83 (0.92)	
Time test statistics		67.60	
Significance		< 0.001	

## Discussion

Based on the results, the level of pain, burning sensation, and OLP lesion decreased in both Curcumin and Prednisolone groups and there was no significant difference between them.

Lichen planus is T cell-mediated autoimmune and an inflammatory disease that affects mucocutaneous. Antigen presentation to CD4+ helper T cells results in the production of cytokines and the activation of CD8+ cytotoxic lymphocytes.

Different amounts of free radicals and reactive oxygen species (ROS) are induced during this process. These reactions can damage lipids, proteins, and nucleic acids in cells [12, 22]. OLP (as a potentially malignant disorder) symptoms, along with its classification, mandates effective management and regular monitoring [22].

As severe pain exists in OLP erosive and atrophic forms with greatly intolerance to hot or spicy food, and these forms may be associated with higher malignancy risk than other forms of OLP [23], they are included in the current study.

Despite many studies conducted to find an effective approach for managing OLP, the results have mostly been unsatisfactory [23]. Although topical Corticosteroid is the first choice in OLP management, it is associated with several adverse effects, such as atrophy of oral mucosa, candidiasis, and tachyphylaxis [23]. In prolonged treatment with local corticosteroids, systemic adverse effects of ocular, endocrine, and metabolic may occur [24].

Many studies have been performed to find an alternative treatment [12].

Curcumin has been shown to exhibit antioxidant, anti-inflammatory, antimicrobial, and anticarcinogenic activities [25]. Given the numerous benefits of Curcumin in treating of lichen planus over steroids, several studies have been done in the past.

The results of case-control studies demonstrated that topical treatment with Curcumin would improve lesions and reduced pain severity similar to triamcinolone cream [2, 17].

The results of other studies demonstrated that oral lesion recovery rate in the OLP patients treated with Prednisone and Curcumin was higher than those received Prednisone alone [19, 26]. Based on the further research results, the group with Curcumin in three doses of 2000 mg per day and for 14 days demonstrated a noticeable recovery in clinical signs and symptoms in comparison with the control group [27]. Chainani et al. conducted another research to evaluate the effect of the treatment duration increase with Curcumin on improving OLP symptoms. The patients were treated with an average dose of 2134.5 Curcumin per day and for 30 months. Sixty percent of the patients showed symptom relief, 35% of them did not trust the results, and 5% of the patients reported that Curcumin did not cause any symptom relief [18]. Patil et al. reported that doses higher than Curcumin 6000 mg perfectly controlled the OLP clinical symptoms, and diarrhea was recognized as one of the side effects of Curcumin dosage [28]. These results are in line with those of the present study.

Curcumin has limits in water solubility and bioavailability due to its hydrophobic nature, challenging Curcumin’s clinical translation into a practical therapeutic agent. Nanoparticles increase the dissolution rate of the hydrophobic agents by supplying a large surface-to-volume ratio [29].

Since, in the current study, Curcumin was Nano-Curcumin, a dose of 80 mg was used, which was significantly less than the dose in other studies using non-nano silic forms. In vivo study showed that low-dose (20 mg/kg) Nano-Curcumin has an equivalent therapeutic effect as high-dose (400 mg/kg) pure Curcumin [29].

In the research conducted by Thomas et al., Curcumin 1% gel 3 times a day and Curcumin 1% gel 6 times a day was compared to Triamcinolone cream. All the groups were treated for 3 months; they showed a decrease in burning sensation, redness, and ulcer. However, the triamcinolone group experienced the highest reduction in burning sensation, redness, and an ulcer [9]. The results of this study are not in line with ours. This dissimilarity can be justified by referring to the different types of Curcumin employed. In the present study, Curcumin was used as an oral-systemic capsule (80 mg), while Thomas et al. employed its 1% gel. Also, the Curcumin treatment duration was shorter than ours. It can be concluded that the amount of Curcumin dose is more important than its use duration in improving OLP. Chainani et al. referred to the same point [26].

Another comparison that clarifies the importance of Nano-Curcumin bioavailability in therapeutic effects in the current study is with the research conducted by Amirchaghmaghi et al. OLP patients were randomly treated with oral Curcumin (2000 mg per day) and placebo for 4 weeks. According to the results, no therapeutic effect was considered for Curcumin in the treatment of OLP [20]. Although in his study all the patients were treated by routine OLP treatments, using Dexamethasone 0.5 mg mouthwash and Nystatin 100,000 unit/ml oral suspension. Using routine OLP treatments can improve the patients’ clinical symptoms to the maximum extent achievable by drug therapy and using other therapies can no longer increase the recovery rate.

Further studies with more follow-ups with recurrence rate estimations are recommended to introduce Nano-Curcumin as a new therapeutic agent in OLP.

## Conclusion

The present research results revealed that oral Nano-Curcumin could be used as an alternative treatment for OLP lesions in those who should not take oral Corticosteroids or in the patients who should take Corticosteroids cautiously. Moreover, oral Curcumin could be used for preventing the recurrence of OLP lesions after the treatment and initial control. Further studies are recommended concerning the latter issue.

## Supplementary Information

**Additional file 1.** CONSORT 2010 Flow Diagram.

**Additional file 2.** Table (*)-Pain VAS mean in the intervention and control groups at the examined times. Table (**): A summary of lesion size mean in the two groups at the examined times.

## Data Availability

The datasets used and analyzed during the current study are available from the corresponding author on reasonable request.

## Abbreviations

OLP: Oral lichen planus
PUVA: Psoralen and ultraviolet A
VAS: Visual Analogue Scale
UVA: Ultraviolet A
UVB: Ultraviolet B
WHO: World Health Organization
