# Supplementary material for: Comparison of oral Nano-Curcumin with oral prednisolone on oral lichen planus: a randomized double-blinded clinical trial
Source: BMC Complement Med Ther. 2020 Oct 31;20:328. doi: 10.1186/s12906-020-03128-7 (PMC7603687; doi:10.1186/s12906-020-03128-7)
Supplement: Supplementary file 1 — Additional file 1. CONSORT 2010 Flow Diagram. [file 12906_2020_3128_MOESM1_ESM.doc]

**
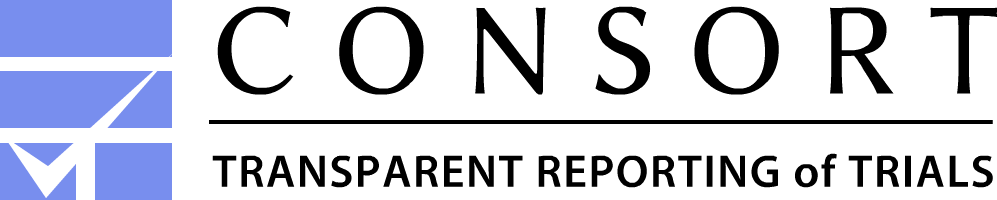
**

**CONSORT 2010 Flow Diagram**

**Allocation**

**Analysis**

**Follow-Up**

**Enrollment**

Assessed for eligibility (n=67)

Excluded (n=7)

♦  Not meeting inclusion criteria (n=4)

♦  Declined to participate (n=3)

♦  Other reasons (n=0)

Analysed (n=29)
♦ Excluded from analysis (give reasons) (n=0)

Lost to follow-up (n=1)

Allocated to Curcumin (n=30)

♦ Received allocated intervention (n=30)

Lost to follow-up (n=2)

Allocated to Prednisolone (n=30)

♦ Received allocated intervention (n=30)

Analysed (n=28)
♦ Excluded from analysis (give reasons) (n=0)

Randomized (n=60)
