# Supplementary material for: Comparison of oral Nano-Curcumin with oral prednisolone on oral lichen planus: a randomized double-blinded clinical trial
Source: BMC Complement Med Ther. 2020 Oct 31;20:328. doi: 10.1186/s12906-020-03128-7 (PMC7603687; doi:10.1186/s12906-020-03128-7)
Supplement: Supplementary file 2 — Additional file 2. Table (*)-Pain VAS mean in the intervention and control groups at the examined times. Table (**): A summary of lesion size mean in the two groups at the examined times. [file 12906_2020_3128_MOESM2_ESM.docx]

Table (*)-Pain VAS mean in the intervention and control groups at the examined times

| Time | Group | Number | Mean (Standard deviation) |
| --- | --- | --- | --- |
| Before the study | Curcumin | 29 | 4.65 (3.39) |
|  | Prednisolone | 28 | 4.89 (3.34) |
| Week 1 | Curcumin | 29 | 4.38 (3.03) |
|  | Prednisolone | 28 | 4.67 (3.45) |
| Week 2 | Curcumin | 29 | 3.41 (2.74) |
|  | Prednisolone | 28 | 3.28 (2.74) |
| Week 4 | Curcumin | 29 | 2.69 (2.89) |
|  | Prednisolone | 28 | 2.33 (2.03) |

Table (**): A summary of lesion size mean in the two groups at the examined times

| Time | Group | Number | Mean  (Standard deviation) |
| --- | --- | --- | --- |
| Before the study | Curcumin | 29 | 3.83 (1.17) |
|  | Prednisolone | 28 | 3.61 (0.98) |
| Week 1 | Curcumin | 29 | 3.48 (1.27) |
|  | Prednisolone | 28 | 3.22 (1) |
| Week 2 | Curcumin | 29 | 2.79 (1.15) |
|  | Prednisolone | 28 | 2.56 (0.92) |
| Week 4 | Curcumin | 29 | 2.34 (1.14) |
|  | Prednisolone | 28 | 1.83 (0.92) |
